# A case report of an unusual non-mucinous papillary variant of CPAM type 1 with KRAS mutations

**DOI:** 10.1186/s12890-020-1088-z

**Published:** 2020-02-24

**Authors:** Timco Koopman, Bart L. Rottier, Arja ter Elst, Wim Timens

**Affiliations:** 1 Department of Pathology, Pathologie Friesland, Leeuwarden, The Netherlands; 2 Department of Pathology and Medical Biology, University of Groningen, University Medical Center Groningen, Groningen, The Netherlands; 3 Department of Pediatrics, University of Groningen, University Medical Center Groningen, Groningen, The Netherlands

**Keywords:** Congenital pulmonary airway malformation (CPAM), Congenital lung disorder, Mucinous adenocarcinoma, KRAS mutation

## Abstract

**Background:**

congenital pulmonary airway malformation (CPAM) is the most frequent congenital lung disorder. CPAM type 1 is the most common subtype, typically having a cystic radiological and histological appearance. Mucinous clusters in CPAM type 1 have been identified as premalignant precursors for mucinous adenocarcinoma. These mucinous adenocarcinomas and the mucinous clusters in CPAM commonly harbor a specific KRAS mutation.

**Case presentation:**

we present a case of a 6-weeks-old girl with CPAM type 1 where evaluation after lobectomy revealed a highly unusual complex non-mucinous papillary architecture in all cystic parts, in which both mucinous clusters and non-mucinous papillary areas harbored the known KRAS mutation.

**Conclusions:**

we found that a KRAS mutation thought to be premalignant in mucinous clusters only, was also present in the other cyst lining epithelial cells of this unusual non-mucinous papillary variant of CPAM type 1, warranting clinical follow-up because of uncertain malignant potential.

## Background

Congenital pulmonary airway malformation (CPAM) is the most frequent congenital lung disorder. Of the five subtypes, CPAM type 1 is the most common. CPAM type 1 typically has a cystic radiological and histological appearance. Because of lung infection risk, CPAM-afflicted lobes are surgically removed after birth. Additionally, microscopic mucinous clusters have been identified as premalignant precursors for mucinous adenocarcinoma. These mucinous adenocarcinomas and the mucinous clusters in CPAM commonly harbor a specific KRAS mutation. We present a case of CPAM type 1 with a highly unusual complex non-mucinous papillary architecture, in which both mucinous clusters and non-mucinous papillary areas harbored the known KRAS mutation.

## Case presentation

### Clinical presentation

**Fig. 1:**
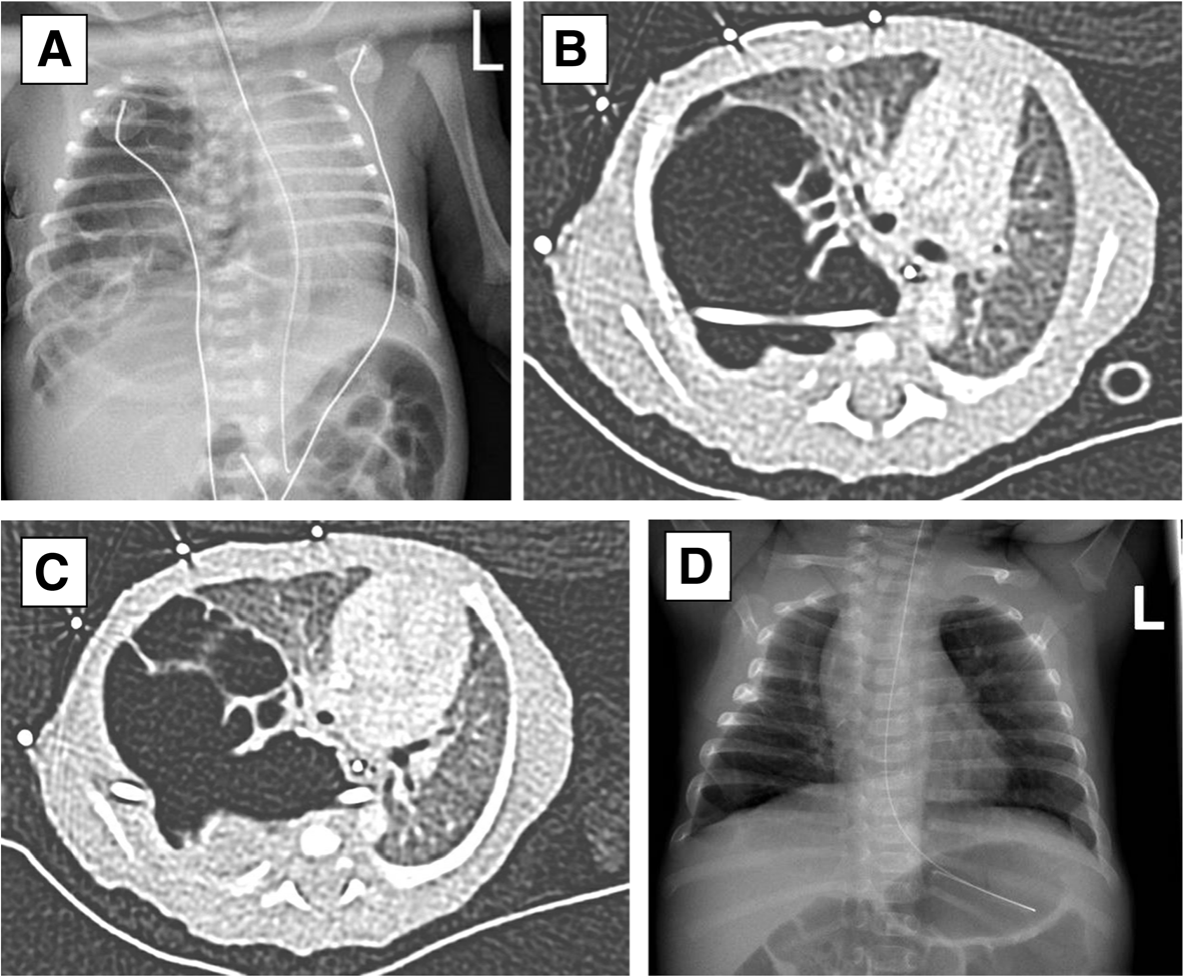
Chest X-ray after initial stabilization on the NICU ward **a** showed a large right lung with cyst-like appearance and a mediastinal shift to the left. High resolution chest CT scans **b** and **c** showed a thick walled multicystic air filled process with a left-sided mediastinal shift. The chest drain can be seen. Chest X-ray at the age of three months **d** showed slight post-operative thoracic cage deformity on the right, a clip in the right hilum, and normal aeration of both the right and the left lung

On the regular 20-week fetal anomaly ultrasound of a female infant, a macrocystic pulmonary malformation of the right lung was found, interpreted as likely congenital pulmonary airway malformation (CPAM). At 23 + 4 weeks of gestation, placement of a shunt was indicated because of a mediastinal shift. Decompression of the left lung was achieved by a shunt in the cysts draining into the amnion. The infant was born spontaneously after 30 + 1 weeks of gestation, with a birthweight of 1.6 kg. At birth, the shunt was dislocated and being cyanotic and bradycardic the girl had to be mechanically ventilated. Initially this was successful, but after six hours chest drainage was indicated again. Four days later the CT-scan showed a multicystic malformation in the right lower lobe, in line with the initial suspicion of CPAM, without abnormal vascular supply. X-rays and CT scans are shown in Fig. 1. Six weeks after birth, when the infant reached the weight of two kilograms, lobectomy of the affected lobe was performed.

### Pathology

**Fig. 2:**
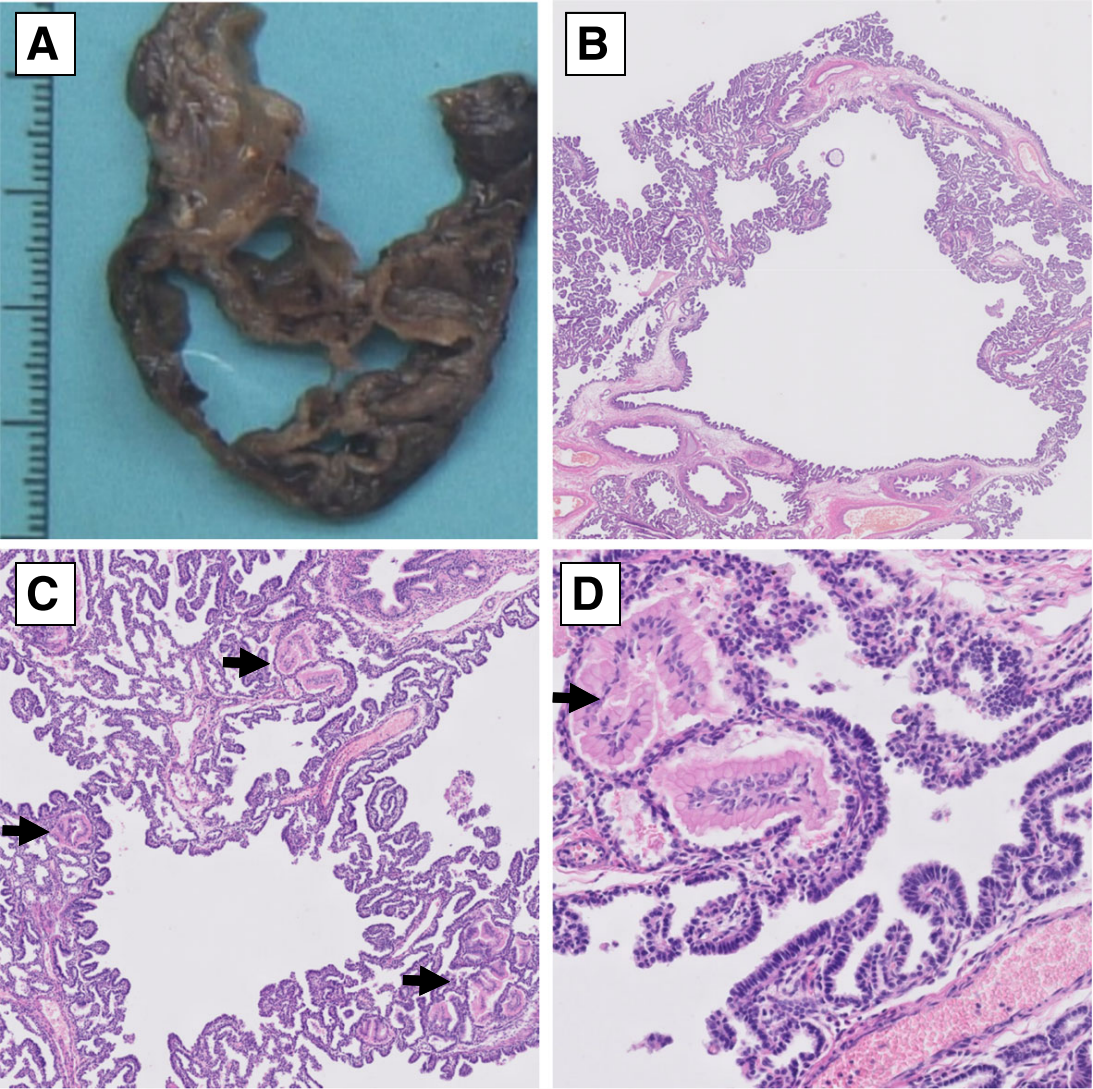
Gross examination showed a cystic lung **a**, with numerous complex non-mucinous papillary projections on histologic evaluation **b-d**. Numerous mucinous clusters were identified **c** and **d**, arrows. Both these mucinous clusters as well as the papillary areas harbored KRAS c.35G > A mutations. Histology images at 10x **b**, 50x **c** and 200x **d** magnification

The pathology findings are shown in Fig. 2**.** Gross examination showed a cystic lobe with multiple cysts larger than 2 cm, the largest more than 3 cm. Microscopic evaluation revealed cysts consistently lined with numerous complex non-mucinous papillary projections, lined with uniform non-ciliated cuboidal to low-columnar epithelium with immature morphology and without cytonuclear atypia, but without ciliated bronchial type epithelial cells. Immunohistochemistry showed diffuse strong positivity of TTF-1 (Thyroid Transcription Factor-1) and Napsin A in the epithelial cells of the papillary structures. Several interspersed small mucinous Periodic Acid Schiff stain (PAS) positive clusters were identified throughout the lesion. Additionally, the PAS stain showed glycogenosis in the alveolar septa in the non-involved part of the lobe, consistent with (variable or possibly resolving) pulmonary interstitial glycogenosis, which can be seen in relatively immature lung tissue [1]. No morphological atypical or malignant foci were found. The lesion was classified as a papillary variant of CPAM type 1 because of the size of the cysts and the architecture of the cyst wall with presence of a smooth muscle layer, with the notion of the unusual non-mucinous papillary morphology.

### Molecular analysis

Molecular analysis was performed on three carefully selected areas: firstly the mucinous clusters, secondly the papillary areas and thirdly the pre-existent normal appearing lung. These areas were manually annotated by the pathologist on the corresponding Hematoxylin and Eosin (H&E) slides, to ensure that the right areas were sampled for molecular analysis. DNA extracted (COBAS FFPE preparation kit, Roche) from Formalin-fixed, Paraffin-embedded (FFPE) material of the three selected areas was sequenced with Next Generation Sequencing (NGS). Library preparation was performed using a custom Ampliseq panel, which included the hotspots of KRAS (codon 12, 13, 61, 117 and 146). The library was sequenced on an Ion Personal Genome Machine™ (PGM™) System (Ion Torrent™). Analysis of the NGS data was performed with JSI Nextseq software (JSI medical systems). Sequencing showed a KRAS: c.35G > A p.(G12D) mutation in the mucinous areas (20% neoplastic cells, variant allele frequency 32%) as well as in the papillary areas (80% neoplastic cells, variant allele frequency 20%). To verify the absence of the KRAS mutation in normal appearing lung, a ddPCR using the KRAS screening assay (ddPCR™ KRAS G12/G13 Screening Kit #1863506, Biorad) was performed. The KRAS mutation was not present in normal appearing lung (limit of detection 2%).

### Follow-up

After surgery there were no per-operative or post-operative complications. Post-operative control X-rays showed a cystic residue, for which follow-up was indicated. During follow-up at the age of 6 months the child was thriving. In a multidisciplinary setting it was agreed upon that based on the potential pathogenicity of the mutations and the fact that the affected lung tissue was completely removed, high resolution chest CT scans will be performed at the ages of 1 and 3 years (arbitrarily chosen), combined with clinical follow-up.

## Discussion and conclusions

A congenital thoracic malformation (CTM), postnatally diagnosed as a congenital pulmonary airway malformation (CPAM) and formerly called congenital cystic adenomatoid malformation (CCAM), is the most frequent congenital disorder of the lung accounting for 25% of all congenital lung disorders [2]. EUROCAT data report a prevalence of 1.05 per 10,000 pregnancies [3]. There are five subtypes of CPAM, depending on typical clinical and histological features [2, 4]. CPAM type 1 occurs most frequently (60–70% of cases) and presents as one or more medium to large cysts measuring more than 2 cm, usually limited to one lobe (95%). It is primarily diagnosed in the first month of life. Because of the risk of lung infections, CPAM-afflicted lobes are surgically removed after birth. Histologic examination typically shows thin-walled cysts lined with ciliated pseudostratified columnar epithelium [2]. These cysts can have polypoid folds [5]. In the present case, histology revealed a highly unusual complex non-mucinous papillary architecture in which the papillary projections were not lined with bronchial epithelial ciliated cells as usual. To date, this has not been described in literature. Fisher et al. described a CPAM type 1 case with papillary architecture [6]. However, they found large papillary projections in an otherwise conventional CPAM and not the complex papillary morphology as seen in the current case.

The use of elective surgery in non-respiratory compromised children remains controversial [7]. Long-term survival after surgical removal of CPAM afflicted lobes is usually very good. However, CPAM type 1 has been associated with the development of mucinous adenocarcinoma (formerly called mucinous bronchioloalveolar carcinoma) [4, 5]. Mucinous clusters are present in one third of CPAM type 1 cases and have been identified as premalignant precursors for mucinous adenocarcinoma, although the occurrence of carcinomatous transformation is only < 1% [5, 8, 9]. In mucinous adenocarcinoma a specific KRAS mutation is found: KRAS c.35G > A, p.(G12D) [10]. This KRAS mutation has also been found in the mucinous clusters in CPAM [11], possibly being premalignant precursors, and was also present our case. Remarkably, the KRAS c35G > A mutation was also found in the other, histologically benign appearing, non-mucinous papillary areas. The clinical relevance of the presence of this mutation in this setting is unclear, but is likely also to be considered as indicating potential malignancy.

In conclusion, we present a case of a 6-weeks-old girl where lobectomy showed a CPAM type 1-like cystic lesion with a highly unusual papillary morphology, in which both mucinous clusters as well as non-mucinous papillary areas harbored a KRAS c.35G > A mutation, in adults known to be associated with adenocarcinoma. Importantly, we found that a KRAS mutation thought to be premalignant in mucinous clusters only, was also present in the other cyst lining epithelial cells of this unusual non-mucinous papillary variant of CPAM type 1, warranting clinical follow-up because of uncertain malignant potential.

## Data Availability

All data generated or analyzed during this study are included in this published article.

## Abbreviations

CCAM: Congenital cystic adenomatoid malformation
CPAM: Congenital pulmonary airway malformation
CTM: Congenital thoracic malformation
FFPE: Formalin-fixed, Paraffin-embedded
PAS: Periodic acid schiff
TTF-1: Thyroid transcription factor 1
